# The interaction between the gut microbiota and dietary carbohydrates in nonalcoholic fatty liver disease

**DOI:** 10.1038/s12276-021-00614-x

**Published:** 2021-05-20

**Authors:** Grace Park, Sunhee Jung, Kathryn E. Wellen, Cholsoon Jang

**Affiliations:** 1 Department of Biological Chemistry, Chao Family Comprehensive Cancer Center, University of California Irvine, Irvine, CA USA; 2 Department of Cancer Biology, University of Pennsylvania Perelman School of Medicine, Philadelphia, PA USA

**Keywords:** Metabolic syndrome, Metabolic syndrome

## Abstract

Imbalance between fat production and consumption causes various metabolic disorders. Nonalcoholic fatty liver disease (NAFLD), one such pathology, is characterized by abnormally increased fat synthesis and subsequent fat accumulation in hepatocytes^1,2^. While often comorbid with obesity and insulin resistance, this disease can also be found in lean individuals, suggesting specific metabolic dysfunction^2^. NAFLD has become one of the most prevalent liver diseases in adults worldwide, but its incidence in both children and adolescents has also markedly increased in developed nations^3,4^. Progression of this disease into nonalcoholic steatohepatitis (NASH), cirrhosis, liver failure, and hepatocellular carcinoma in combination with its widespread incidence thus makes NAFLD and its related pathologies a significant public health concern. Here, we review our understanding of the roles of dietary carbohydrates (glucose, fructose, and fibers) and the gut microbiota, which provides essential carbon sources for hepatic fat synthesis during the development of NAFLD.

## Fatty liver disease: A conspiracy of carbohydrates and gut microbes

Evidence is emerging of a role for interactions between gut microbes and dietary carbohydrates in non-alcoholic fatty liver disease (NAFLD), one of the most prevalent liver disorders of adults worldwide, with children and adolescents also increasingly affected. NAFLD can progress into even more serious conditions such as steatohepatitis, cirrhosis, liver cancer and liver failure. Researchers led by Cholsoon Jang at the University of California Irvine review understanding of the role of dietary glucose, fructose, fiber and gut microbial metabolism in contributing to NAFLD. The involvement of gut microbes in hepatic assimilation of carbohydrates into fatty acids that accumulate in NAFLD indicates possible treatment strategies based on interfering with microbial metabolism. New understanding of the interactions between carbohydrate metabolism and gut microbes will also help reveal why some people develop NAFLD and suggest appropriate preventive interventions.

## Bridging carbohydrate metabolism and hepatic de novo lipogenesis

Carbohydrate and lipid metabolism is highly intertwined in the liver. De novo lipogenesis (DNL), a process that converts dietary carbohydrates into fat, is one such metabolic link. Dietary carbohydrates and lipids follow distinct paths from the gut lumen to other parts of the body. Due to their hydrophobicity, dietary fats, mostly composed of long-chain fatty acids, are packaged as triglycerides (TGs) into chylomicrons in intestinal enterocytes and distributed to nonhepatic tissues (e.g., adipose depots) through the lymphatic system, thereby bypassing metabolism in the liver^5^. On the other hand, dietary carbohydrates absorbed by the small intestine go directly to the liver. When the carbohydrate level reaches a certain threshold, diverse metabolic pathways in hepatocytes are triggered to clear away excess carbohydrates. Glycogen synthesis and DNL are such pathways. The resulting newly synthesized fatty acids are stored in hepatocytes as lipid droplets, released into the bloodstream as lipoprotein particles (e.g., very-low-density lipoproteins or VLDLs) to feed other organs, or oxidized in the liver when other energy sources are scarce (e.g., under fasting conditions). Thus, DNL is a key mechanism that contributes to the metabolic flux of dietary carbohydrates into lipids, especially in the postprandial state and under disease conditions^6,7^.

**Fig. 1:**
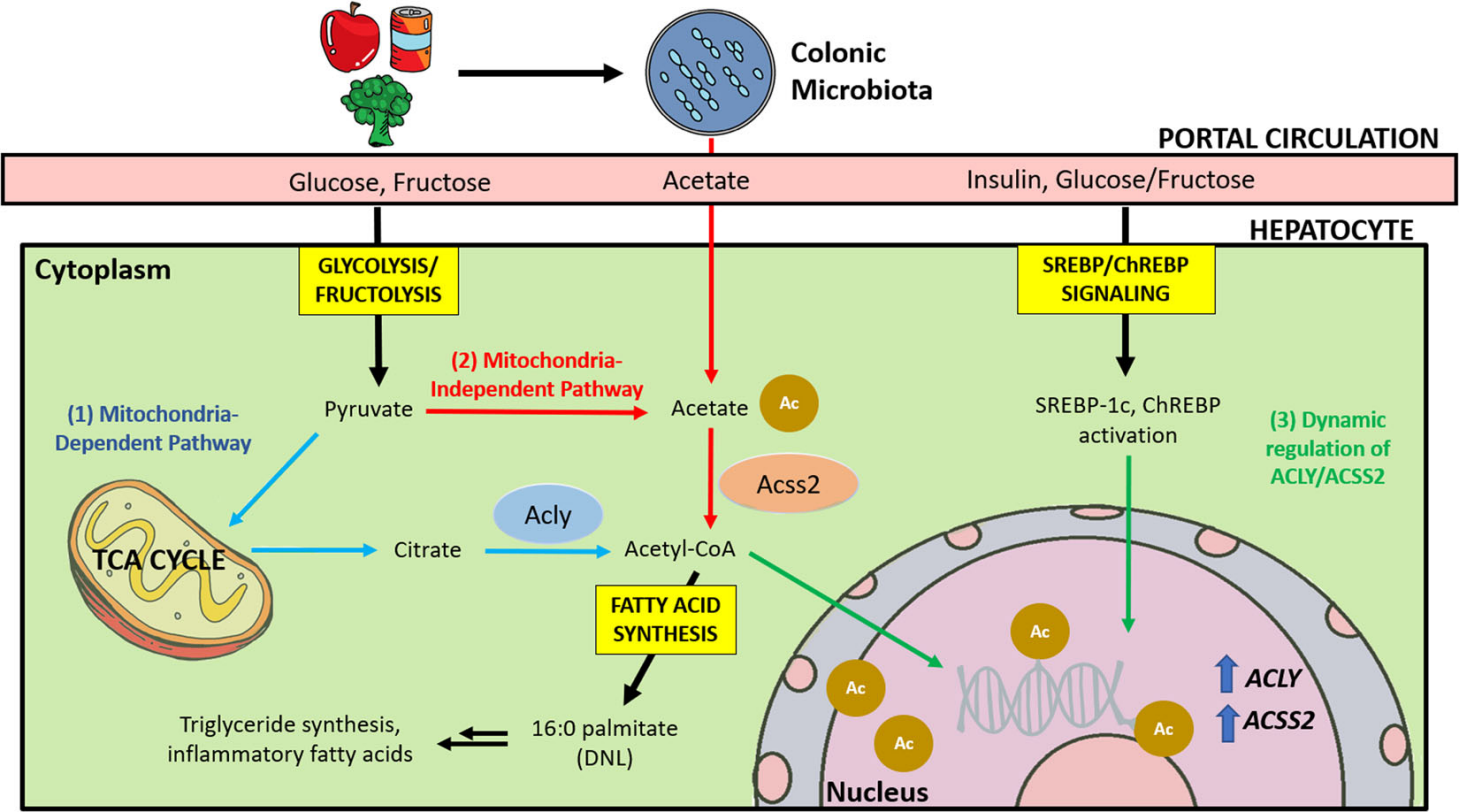
ACLY- and ACSS2-mediated production of lipogenic acetyl-CoA for DNL. Dietary carbohydrates, such as glucose and fructose, are absorbed by the small intestine and delivered to the liver via the portal circulation. Alternatively, fructose or fibers reach the colon and are catabolized by the gut microbiota, producing short-chain fatty acids, including acetate. In hepatocytes, glycolysis/fructolysis provides carbon sources for ACLY-mediated generation of cytosolic acetyl-CoA from cytosolic citrate. On the other hand, acetate provides carbon for ACSS2-mediated synthesis of cytosolic acetyl-CoA, especially in low-energy environments. Acetyl-CoA can also be used for histone acetylation in the nucleus. Cytosolic acetyl-CoA is used for DNL. Metabolites of glucose or fructose also act in a signaling capacity to turn on lipogenic transcription factors, such as SREBP-1c and ChREBP, a process that is further augmented by insulin signaling.

DNL is controlled by allosteric regulation of diverse lipogenic enzymes. Dietary carbohydrates (mainly glucose) first enter glycolysis in the cytosol and become pyruvate, the terminal product of glycolysis (Fig. 1). Pyruvate then either becomes lactate or enters the mitochondria for further oxidation in the citric acid (TCA) cycle. In high-energy states, citrate is transported back to the cytosol and converted into cytosolic acetyl-CoA by ATP citrate lyase (ACLY)^8^. This generation of cytosolic acetyl-CoA by ACLY is the key step in lipogenesis, as mitochondrial acetyl-CoA cannot directly participate in DNL. Notably, in obese individuals, a small fraction of short-chain fatty acids can be transported from the mitochondria to feed DNL, but the biological significance of this pathway needs more investigation^9^. Acetyl-CoA carboxylase (ACC) then generates malonyl-CoA from cytosolic acetyl-CoA, the commitment step in DNL. This process is allosterically promoted by cytosolic citrate. Importantly, malonyl-CoA serves as a potent allosteric inhibitor of fatty acid β-oxidation. This mechanism is thought to act as a metabolic safeguard against wasteful energy expenditure from simultaneous fatty acid synthesis and oxidation^10^. The final step of DNL is the conversion of malonyl-CoA into palmitate (16:0) by fatty acid synthase (FAS). Other processes, such as fatty acid elongation and desaturation, also occur after palmitate is synthesized^7,10^.

Another crucial layer of DNL regulation occurs at the transcriptional level, which is in part hormonally controlled. Under postprandial conditions, the release of insulin stimulates the expression of lipogenic genes in the liver to promote DNL. Sterol regulatory element-binding protein (SREBP) is a well-known transcription factor that simultaneously induces many lipogenic genes. While there are several SREBP isomers, SREBP-1c is particularly insulin-sensitive compared to the other SREBP isoforms SREBP-1a and SREBP-2^11–14^. In mice, the loss of *Srebp-1c* resulted in failure to induce DNL enzymes, including FAS and ACC, in response to postprandial increases in insulin levels, and this lack of response was only partially compensated by SREBP-1a/SREBP-2^15^. Therefore, SREBP-1c is the primary isoform responsible for insulin-induced DNL in hepatocytes. The other SREBP isoforms have been linked to separate metabolic pathways; SREBP-2 is associated with cholesterol synthesis (another important feature of lipid metabolism), while SREBP-1a is mainly expressed in the spleen^16,17^.

In addition to insulin, full activation of the hepatic lipogenic program requires glucose^18^. While glucose itself has not been found to modulate transcriptional DNL activity, one glycolytic metabolite, glucose-6-phosphate (G6P), has been linked to increased *FAS* and *ACC* expression^19,20^. This result was further substantiated by in vitro analysis of *Fas* expression and glucokinase activity, the enzyme that converts glucose into G6P. Both glucose and glucokinase were found to be required to induce *Fas* expression in response to insulin^20^. Glucose-induced DNL proceeds through the upregulation of both glucokinase and SREBP-1c in response to insulin^14,21^. Dentin et al. further expanded on this model and implicated carbohydrate responsive element-binding protein (ChREBP) as another important transcription factor regulated by G6P to trigger DNL^22^. However, this link between ChREBP and dietary carbohydrates is not exclusive to glucose; hepatic ChREBP is particularly sensitive to fructose (see more details in the section below)^23^. Indeed, G6P may not be the only metabolite that stimulates ChREBP activity, and various other metabolites (e.g., xylulose 5-phosphate, 3-carbon glycolytic metabolites) have also been suggested to have this effect^23–25^.

Importantly, *ChREBP* knockdown decreased the effects of glucose on both lipogenic and glycolytic gene induction in response to insulin^22,24^. It is thus evident that ChREBP mediates insulin-glucose synergy^26^. One mechanism of action involves glucose-mediated increases in *ChREBP* expression, nuclear entry, and binding to the carbohydrate response element (ChoRE), which in turn induces not only lipogenic genes but also SREBP-1c^27,28^. ChREBP and SREBP thus rely on each other and work synergistically as follows: 1) ChREBP relies on activation by glycolytic intermediates, which, in turn, are generated by genes controlled by SREBP-1c^22^. 2) Meanwhile, the deletion of hepatic *Chrebp* reduces SREBP-1c levels^29^. 3) Furthermore, overexpression of hepatic *Chrebp* does not increase postprandial lipogenesis when *Srebp-1c* is lacking^29^.

Collectively, maximum activation of DNL requires concurrent upregulation of lipogenic and glycolytic gene expression, for which glucose and insulin are necessary to stimulate lipogenic transcription factors. In this regard, for insulin-resistant individuals with NAFLD, increases in both circulating glucose and insulin can be synergistic drivers of continuously elevated DNL, the hallmark of this disease^18^.

## Fructose metabolism both triggers and feeds DNL

In addition to glucose, fructose is another major dietary carbohydrate in modern society that is closely linked to NAFLD/NASH development. While fructose and glucose have the same molecular formula (C_6_H_12_O_6_), they are metabolized by organs quite differently and have divergent effects on hepatic DNL^30^. For example, fructose can serve as a preferential substrate of DNL due to the faster rate of fructolysis compared to that of glycolysis^31–33^. During the primary steps of glycolysis, hexokinase and phosphofructokinase are allosterically regulated, suppressing excessive glycolysis. In contrast, ketohexokinase-c (KHK-C), the primary fructolytic enzyme, has constitutively high activity and thus bypasses these rate-limiting glycolytic steps. This results in rapid fructose catabolism, which triggers ATP depletion and carbon entry into the TCA cycle as well as DNL^34^. However, most in vitro hepatocyte studies used supraphysiological millimolar concentrations of fructose in the culture medium. Isotope tracing studies did not carefully differentiate between the direct and indirect contributions of fructose to the hepatic DNL pathway.

This raised the possibility that fructose-dependent DNL and NAFLD may also be linked to nonhepatic fructose catabolism. Supporting this notion, high-fructose feeding was shown to increase hepatic steatosis and inflammation in both wild-type and liver-specific *Chrebp*-knockout mice, which exhibit blunted hepatic fructose metabolism^23^. Recently, it was shown that the small intestine actively catabolizes fructose in a KHK-dependent manner, thereby reducing the amount of fructose that reaches the liver and preventing hepatic steatosis^30,35^. Remarkably, isotopic tracing of fructose in various genetically modified mouse models demonstrated that some fructose-derived DNL carbon sources in the liver are not derived directly from fructose itself but rather derived indirectly from fructose-derived acetate made by the gut microbiota^36,37^. These new data challenge the previously known role of fructose-derived direct lipogenesis in hepatocytes and emphasize the complex interaction between the intestine, liver, and gut microbiota.

What is the pathological role of fructose catabolism in the liver, then? Accumulating evidence has indicated that fructose is an especially potent inducer of hepatic DNL signaling. For example, ChREBP is more strongly responsive to fructose than glucose^38^. Both nuclear SREBP-1 levels and ChREBP activity were increased in the liver after fructose feeding but not glucose feeding^39^. Mice lacking whole-body or hepatic KHK, but not intestinal KHK, showed protection from fructose-induced hepatic steatosis^40–42^, demonstrating that hepatic fructose catabolism is a key molecular event that triggers NAFLD.

In humans, chronic high-fructose feeding in male participants increased both postprandial and steady-state serum TG levels-effects that were not seen in glucose-fed individuals^43^. Bolus fructose consumption also induced increased palmitate incorporation into hepatic lipoproteins as well as increased postprandial production of TGs in fructose-fed, but not glucose-fed, individuals^44^. *KHK-C* overexpression has been commonly observed in obese NASH patients^45^, supporting the notion that it is a proximal step of fructose metabolism needed to provide lipogenic signals to induce pathogenesis. These strong genetic mouse data in conjunction with human epidemiological and feeding studies provide promising pharmaceutical basis for the clinical use of KHK inibitors in fatty liver treatment^46^.

## Causes and comorbidities of NAFLD and NASH

### Hepatic DNL

The hallmark of NAFLD is the accumulation of lipids in the liver. What is the major source of these lipids? Early research in lean, fasted individuals suggested that DNL makes only a minimal contribution to hepatic lipid levels, as most hepatic lipids were traced back to fatty acids released from adipose tissue. However, further studies with sophisticated isotope tracing have shown that DNL plays a larger role in NAFLD patients than previously suggested^47–49^. Comparison of NAFLD patients’ livers with high versus low fat accumulation reported a 3.5-fold increase in DNL in patients with high fat accumulation but no difference in the contribution of dietary or free fatty acids to the hepatic TG pool^47^. Fabbrini et al. also observed that a significant portion of serum free fatty acids were not incorporated into hepatic lipid stores in obese individuals with steatosis, suggesting that these lipids did not originate from free fatty acids but rather originated from DNL^49^. NAFLD patients also exhibited strongly upregulated expression of DNL enzymes, such as ACC1, ACC2, and FAS^50,51^. Interestingly, NAFLD patients exhibited increased DNL even during fasting^52^, indicating the lack of metabolic flexibility in these patients as opposed to the robust suppression of DNL during fasting observed in healthy individuals^44,53^. This may also reflect other metabolic inflexibilities, such as insulin resistance, typically found in NAFLD patients.

While upregulated DNL is a key feature of NAFLD, it may not be the sole instigator of disease progression. Like their non-NAFLD counterparts, NAFLD patients still derive the majority of their hepatic TG stores from fatty acids released by adipocytes;^52,54,55^ 26% of hepatic TGs were found to come from fatty acids synthesized de novo, whereas ~60% came from serum fatty acids and ~10% from diet^52^. Thus, in addition to upregulated DNL, other critical factors induce disease progression to NASH, as discussed below.

### Hepatic inflammation and insulin resistance

Mild cases of NAFLD can be asymptomatic or benign. However, progression into NASH is characterized by inflammation, hepatocyte apoptosis, and steatosis. One longitudinal study found that individuals with NASH, but not those with only NAFLD, showed increased mortality and the increased occurrence of end-stage liver disease than the general population^56,57^. These results were further corroborated by Dam-Larsen et al., who concluded that a large population of individuals with simple NAFLD had a favorable long-term prognosis with no disease progression^58^. However, co-diagnosis with other metabolic diseases, such as type 2 diabetes, were correlated with higher rates of cirrhosis, end-stage liver disease, and death^59^. In a cohort of NAFLD patients without steatohepatitis, cardiovascular disease (CVD) and cancer were found to be the leading causes of mortality two decades after initial diagnosis^58^. It is possible that this proclivity for CVD over cirrhosis and end-stage liver disease is a result of systemic metabolic disorder and dysfunction, as NAFLD is often also co-diagnosed with insulin resistance, obesity, and hyperlipidemia^60^. Thus, the lethality of NAFLD may be due to hepatic manifestation of a systemic metabolic syndrome rather than the direct result of damage by steatosis.

Systemic insulin resistance is a key component in NAFLD, as patients have decreased insulin sensitivity across adipocytes, hepatocytes, and skeletal muscle^61^. A body of longitudinal human studies also suggests that steatosis contributes to the development of insulin resistance. Ekstedt et al. found that 78% of NAFLD patients developed impaired glucose tolerance or type 2 diabetes after their initial diagnosis^57^. Cohort studies of East Asian men corroborated these results and established NAFLD as a risk factor for type 2 diabetes, even in nonobese individuals^62,63^. Accumulated hepatic lipids, formed either by DNL or from free fatty acids, have been linked to the onset of hepatic insulin resistance. A positive correlation between high hepatic/visceral fat and hepatic insulin resistance was found in NAFLD patients^64,65^. In rats, 2,4-dinitrophenol (DNP), a drug that promotes β-oxidation in hepatocytes, reduced the hepatic lipid content and improved insulin sensitivity^66^. Conversely, systemic insulin resistance can cause hepatic lipid accumulation in NAFLD. Insulin-resistant adipocytes have higher rates of lipolysis, releasing excessive free fatty acids into the circulation that are incorporated into liver lipids. Another insulin-mediated mechanism is DNL upregulation. Glucose uptake in skeletal muscle and adipose tissue is dependent on insulin, while in hepatocytes, glucose uptake is largely insulin-independent^67^. Thus, this excessive influx of glucose into hepatocytes can cause steatosis via DNL^68^.

However, it is noteworthy that hepatic lipid accumulation does not always share a causal relationship with insulin resistance. Overexpression of diacylglycerol acyltransferase 2 (DGAT2), an important enzyme in the final production of TG synthesis, raised hepatic TG levels and caused steatosis but did not result in hepatic or systemic insulin resistance^69^. This finding indicates that the quality of accumulated lipids rather than their quantity matters, as further discussed below.

### Lipotoxicity

As previously mentioned, not every case of NAFLD leads to chronic inflammation, hepatocyte apoptosis, and cirrhosis. As such, there must be a metabolic distinction between individuals with steatosis and those with progressive degeneration. The development of NASH and cirrhosis from NAFLD has been described by the “multiple-hit” hypothesis. The “first hit” of insulin resistance leads to lipid accumulation in the liver, which makes the organ more susceptible to the secondary “multiple hits” of oxidative stress and inflammation^70^. In this model, the accumulation of TGs is thought to dysregulate lipid flux throughout the liver in this “first hit”. However, Listenberger et al. suggested that TG formation may be a form of protection against lipotoxicity from free fatty acids^71^. The results from a similar study with obese *Dgat2*-knockout mice with NASH also support the findings of Listenberger et al., as a decrease in hepatic TG stores failed to dampen oxidative stress signals or NASH progression^72^.

Even more than the level of fatty acid influx to the liver, the nature of these fatty acids may contribute to lipotoxicity. A higher ratio of saturated fatty acids (SFAs) to monosaturated fatty acids (MUFAs) has been connected to NASH development^73,74^. The conversion of lipotoxic SFAs to MUFAs is mediated by stearoyl-CoA desaturase-1 (SCD1). Free fatty acid influx upregulates *SCD1* expression, resulting in MUFA formation, TG storage, and ultimately, liver adaptation with no progression beyond steatosis. However, *SCD1* downregulation triggers mitochondria-mediated apoptosis through death receptor signaling, ER stress, and the release of proteases in the cytoplasm from increased lysosomal permeability^74^. Identification of key lipotoxic lipid species (e.g., ceramides, diacylglycerol, SFAs, acylcarnitine, lipopolysaccharides) and associated metabolic and signaling pathways is thus crucial to prevent disease progression from NAFLD to NASH.

### Obesity

While some NAFLD patients are lean, obesity is still prevalent in individuals with NAFLD^58^. In obese individuals with type 2 diabetes, regulation of adipocyte lipolysis is largely absent, which promotes the release of fatty acids into the circulation. An increase in fat deposits, particularly an increase in the mass of visceral adipose tissue, further contributes to the influx of free fatty acids into the liver^60,75^. Yamaguchi et al. found that the suppression of TG production failed to stop liver damage or fibrosis in obese mouse models, suggesting that increased adipocyte lipolysis and the resultant influx of fatty acids into the liver make a large contribution to NASH pathologies^72^. Quantitatively, the distribution of visceral fat in the body is marginal compared to the abundance of subcutaneous fat. However, large visceral deposits in obese women have been linked to suppression of the antilipolytic effect of insulin, and increased levels of free fatty acids have been found in the hepatic portal in individuals with upper body obesity^75,76^. Although the mechanism by which visceral adiposity selectively contributes to NAFLD remains contentious, the close proximity of visceral fat to the portal circulation may facilitate the delivery of free fatty acids and inflammatory factors (e.g., derived from immune cells in adipose depots) to the liver^77^.

## Key DNL enzymes as potential therapeutic targets for NAFLD treatment

While there are many enzymes in the DNL pathway, only a few of them are altered in NAFLD patients and are potentially targetable for NAFLD treatment without detrimental side effects. Here, among these enzymes, we focus on two crucial lipogenic enzymes that have been extensively studied for therapeutic intervention.

### ACLY

ACLY is a critical enzyme in DNL that converts cytosolic citrate into acetyl-CoA to provide carbon sources for DNL (Fig. 1). Acetyl-CoA then enters both the fatty acid synthesis and mevalonate pathways to produce fatty acids and cholesterols, respectively. ACLY dysregulation was previously implicated in diseases associated with elevated fatty acid and cholesterol synthesis, such as cancer and atherosclerosis^78,79^. *ACLY* silencing, inhibition, and genetic deletion were found to suppress cell proliferation and tumor growth in several preclinical models^80–85^. One recent study on atherosclerosis also found that *Acly* deletion in macrophages stabilized atherosclerotic plaques—a necessary preventative treatment for stroke—and attenuated the ACLY-mediated inflammatory response in the macrophages. These phenotypes were also linked to dysregulated cholesterol and fatty acid synthesis^86^.

Abnormally elevated expression of *ACLY* has been linked to NAFLD in both humans and mice. Dietary carbohydrates (both glucose and fructose) increase the mRNA expression of *Acly* in mice and rats^7,87^. In NAFLD patients, this diet-mediated *ACLY* induction occurs mainly through SREBP-1c^51,88^, but a recent mouse study showed that ChREBP is also responsible for fructose-induced expression of ACLY and other glycolytic, fructolytic, and lipogenic genes^89^. Furthermore, ACLY is an essential contributor to global histone acetylation. Under nutrient-rich conditions, ACLY is the primary enzyme that mediates the production of glucose-derived nuclear acetyl-CoA, the source of histone acetyl groups^80^. ACLY-dependent histone acetylation has also been linked to selective control of key genes involved in glucose usage in adipocytes, including phosphofructokinase (PFK) and glucose transporter 4 (GLUT4)^90^.

While it is evident that *ACLY* is transcriptionally upregulated in NAFLD, posttranslational regulation of ACLY may also be an important contributing factor to disease pathology. ACLY nuclear localization is activated by phosphorylation at residue S455 by protein kinase B (AKT)^91,92^. ACLY is also phosphorylated at this site by protein kinase A (PKA), which significantly increases ACLY enzyme activity (6-fold increase in V_max_ compared to that of nonphosphorylated ACLY)^93^. Recently, BDK, a kinase of branched chain ketoacid dehydrogenase (BCKDH), was shown to phosphorylate ACLY at the same serine residue independent of AKT and is also upregulated by ChREBP^94^. Small molecule-mediated inhibition of BDK improved hepatic steatosis and the insulin response in insulin-resistant Zucker fatty rats. Furthermore, overexpression of BDK increased ACLY phosphorylation and hepatic DNL.

In addition to its phosphorylation, the acetylation of ACLY seems to play important roles in NAFLD. In hepatocytes, acetylation at three lysine residues has been shown to increase ACLY enzyme stability, resulting in constitutive activation of DNL^95,96^. Both mice and humans with NAFLD showed increased levels of not only total ACLY but also acetylated ACLY. Furthermore, while *Acly* knockdown suppressed steatosis in mice fed a high-fat, high-sugar diet, high levels of ACLY 3KQ (mimicking the constitutively acetylated form) induced steatosis more strongly than overexpression of ACLY wild-type^96^. Mechanistically, ACLY acetylation increases enzyme stability by antagonizing its ubiquitylation^95,96^. At least two responsible E3 ligases have been identified to negatively regulate ACLY^97,98^. Overexpression of one of these E3 ligases, HMG-CoA reductase degradation protein (Hrd1), decreased both acetyl-CoA and ACLY-mediated DNL in the hepatocytes of *db/db* and *ob/ob* mice^97^. This effect ameliorated hepatic lipid droplet accumulation and decreased hepatocyte inflammation in vivo. *Hrd1* expression was also negatively correlated with NAFLD in mice, indicating that failure to activate the ubiquitin pathway and the subsequent accumulation of ACLY contribute to NAFLD development. More broadly, the proteosome-mediated pathway seems to prevent inflammation-dependent proteotoxicity in NAFLD^99^.

### Current ACLY therapeutics

Statins, a class of HMG-CoA reductase inhibitors, have traditionally been used to attenuate atherosclerosis by reducing low-density lipoprotein (LDL) cholesterol levels. However, the emergence of small-molecule therapeutics targeting ACLY has presented new treatment options for individuals particularly sensitive to statin-related muscle symptoms or those who require a more robust reduction in LDL cholesterol. Bempedoic acid (BemA/ETC-1002), a recently FDA-approved LDL cholesterol-lowering drug, is one such therapeutic that has been singled out for ACLY inhibition; BemA was found to reduce lipogenic intermediates such as acetyl-CoA, malonyl-CoA, and HMG-CoA while subsequently increasing citrate in rat hepatocytes^100^. In one rodent model, BemA relieved atherosclerotic symptoms and lowered LDL cholesterol and triglyceride levels in ApoE-knockout and ApoE/AMPK β-1 double-knockout mice^101^. These results suggest that BemA regulates lipid metabolism and protects against LDL cholesterol and inflammation regardless of AMPK activation in the liver. As a prodrug, BemA also requires bioactivation by very long-chain acyl-CoA synthetase-1 (ACSVL1), an enzyme primarily found in the liver^102^. This mediation by hepatic ACSVL1 may thus circumvent muscle effects from statin usage. Current data for BemA usage in humans are primarily directed toward the treatment of atherosclerosis over NAFLD; nevertheless, phase III clinical trials have suggested positive outcomes in lowering LDL cholesterol levels when BemA is administered in conjunction with other therapeutics^103^.

### Acetyl-CoA synthetase short-chain family member 2 (ACSS2)

ACLY activity alone clearly does not account for the cell’s entire lipogenic capacity. Cell viability was not significantly impacted in *Acly*-knockout mouse cell lines^80^. Importantly, acetate was found to be a requirement for maintaining viability in *Acly*-knockout cells by supporting cellular acetyl-CoA levels and DNL^80^. Mice heterozygous for *Acly* also did not show fluctuations in lipid concentration or synthesis, suggesting that the lipogenic program proceeds normally at half ACLY protein levels^104^. ACSS2 is one enzyme that may explain this rescue of lipogenic capacity via its involvement in an alternative acetyl-CoA-generating pathway (Fig. 1). Similar to other members of the ACS enzyme family, ACSS2 facilitates the formation of a thioester bond between acetate and CoA to form acetyl-CoA in an ATP-dependent manner.

In *ACLY*-knockout cell lines, the addition of exogenous acetate at physiological blood concentrations sustained acetyl-CoA levels and lipogenic metabolites via *ACSS2* induction, suggesting that circulating acetate is likely adequate for compensatory lipid synthesis^80,105^. Similarly, *ACLY* silencing increased *ACSS2* expression and maintained fatty acid and mevalonate synthesis pathways to preserve the ability of cancer cells to proliferate^81^. In mice, the loss of *Acly* in white adipose tissue strongly induced *Acss2* expression and promoted the contribution of *Acss2* to cellular acetyl-CoA pools via acetate incorporation without any discernable effect on weight^80^ unless mice were fed a high-carbohydrate diet, which provoked a strong dependence on ACLY in adipocytes^106^. In the liver, however, ACSS2 plays a major compensatory role in the context of high-fructose feeding. Hepatocyte-specific *Acly-*KO mice had hepatic TG levels comparable to those of their wild-type counterparts when fed a high-fructose diet^37^.

Despite this apparent metabolic redundancy, there is also evidence that the method of nutrient consumption—particularly, bolus versus gradual fructose feeding—results in differential ACLY and ACSS2 utilization in the liver. When fructose is fed gradually, both ACLY and ACSS2 contribute to lipogenic acetyl-CoA via citrate cleavage and acetate uptake, and suppression of both enzymes is necessary to reduce DNL^37^. However, bolus fructose consumption may overload the absorption capacity of the small intestine, and the resulting fructose spillover to the colon generates copious acetate by the microbiome^30^. This acetate significantly increases hepatic acetyl-CoA pools via ACSS2^37^. The role of the microbiota in DNL and NAFLD will be discussed in a later section.

ACSS2 also mediates acetate usage for other cellular processes, such as energy production and gene regulation^107,108^. During fasting, the liver releases acetate into the circulation by keogenesis from long-chain fatty acids. Induction of ACSS2 increases the usage of serum acetate as a fuel source in organs such as the heart, skeletal muscle, and even cancers^109–113^. Furthermore, similar to ACLY, ACSS2 can translocate into the nucleus and supply acetyl-CoA for histone acetylation to regulate gene expression^108,114^. Takahashi et al. found that this nuclear acetate remains separate from mitochondrial acetate pools^115^, suggesting that most of the acetate involved in histone acetylation is produced within the nucleus. Consistently, isotope labeling of exogenous acetate is very low in acetylated histones, especially in energy-limited environments^108^. Nuclear ACSS2 thus not only provides acetyl-CoA for histone acetylation but also recaptures, reuses, and retains nuclear acetate.

While histone acetylation contributes to global gene regulation^116^, nuclear ACSS2 has been linked to specific modulation of genes involved in lysosomal biogenesis, autophagy, and hippocampal memory^117,118^. In one study, *Acss2* silencing significantly reduced fatty acid synthesis enzymes and made mice resistant to developing hepatic steatosis upon high-fat diet feeding^119^. Interestingly, the authors showed that high-fat diet feeding increased nuclear ACSS2 in intestinal enterocytes, which was correlated with upregulation of lipid absorption genes^119^. The authors thus proposed that ACSS2 coordinates the cell’s adaptation to energy availability through control of genes in lipid metabolism. However, a mechanistic link between nuclear ACSS2 localization and the promotion of lipogenic genes has yet to be established.

Finally, ACSS1, a cousin of ACSS2, may also be relevant to DNL in NAFLD. *ACSS1* is highly expressed in the liver, and its expression is induced by SREBP^120^. Unlike cytosolic or nuclear ACSS2, however, ACSS1 is primarily associated with mitochondria and has been linked to mitochondrial reactions such as fatty acid β-oxidation^121^. Nevertheless, ACSS1 has been suggested to be at least partially functionally redundant to ACSS2 and to play a background role in acetate-derived lipogenesis under hypoxic conditions^113^. Similarly, Gao et al. suggested that both ACSS1 and ACSS2 are necessary for the expression of lipogenic genes, as the knockdown of either ACSS1 or ACSS2 alone did not seem to inhibit histone acetylation^122^.

## Role of the gut microbiota in NAFLD

Given that the liver is directly linked to the intestine via the portal blood and that the gut microbiome is a major source of acetate, it is not surprising that the gut microbiome plays important roles in NAFLD. Sequencing studies indicated specific changes in microbiota composition in NAFLD subjects. Healthy versus obese/NAFLD individuals showed marked population differences in members of the phyla Bacteroidetes and Firmicutes^36,123^. Diets supplemented with high-fructose corn syrup to induce NAFLD resulted in an increased Firmicutes:Bacteroidetes ratio^124,125^. However, a recent study of 10 obese patients found that fructose did not induce microbial changes^126^. Since this study used a solid form of fructose, it is possible that the small intestine efficiently prevented fructose spillover to the colon. Therefore, further studies with other experimental conditions (e.g., a liquid form of fructose, various doses and durations) are required. Most studies that support the causal link between the microbiota and NAFLD have reported the results of fecal transplantation from donors fed NAFLD-inducing diets^127–138^, which are summarized in Table 1.

**Table 1:** Studies suggesting a causal relationship between the gut microbiota and NAFLD.

Ref	Study model	Treatment	Diet	Main results
128	8-week-old male germ-free (GF) C57BL/6 J mice	Received the microbiota from obese human donor before or after a dietary weight loss program	Normal chow diet for 4 weeks	Development of liver steatosis in mice received from donor before dietary weight loss program. High hepatic TGs and total cholesterol in mice received the microbiota from donor before weight loss program.
129	8-week-old male GF C57BL/6 J mice	Received the microbiota from mice developed hyperglycemia or normoglycemia after 16 weeks of high fat diet (HFD)	60% kcal HFD for 16 weeks after colonization.	High hepatic DNL and steatosis, abundant *Barnesiella* and *Roseburia* genera in mice colonized from hyperglycemia mice. No differences in SCFAs, but increased levels of branched-chain fatty acids.
130	8-week-old GF C57BL/6 J mice	Conventionalization with normal mice	Normal chow diet for 4 weeks	High induction of hepatic lipogenesis in conventionalization compared with GF.
131	6 to 8-week-old GF C57BL/6 J mice		39% and 32.8% (wt) high-fat, high-sucrose diet or normal chow diet with 30% fructose water for 8 weeks	Elevated hepatic TG in high fat, sucrose fed group and high fructose-fed group compared with GF.
132	GF C57BL/6 N mice	Antibiotics	Normal chow diet	Desaturated and elongated hepatic long-chain fatty acids in antibiotics treated mice.
133	6-week-old C57BL/6 J mice	Antibiotics	30% fructose water for 8 weeks	Reduced hepatic lipid accumulation in fructose-fed mice treated with antibiotics.
134	8 to 12-week-old male C57BL/6 J mice	Oral gavage of cecum content obtained from chow- or HFD-fed mice with bile duct ligation	60% kcal HFD or normal chow diet for 4 weeks	Reduced ratio between Bacteroidetes and Firmicutes in mice received from HFD. Enhanced liver damage in mice received from HFD.
135	4-week-old male GF C57BL/6 J mice	Inoculation of Bacteroidetes or Firmicutes	60% kcal HFD for 16 weeks	High hepatic lipid, levels of hepatic FAS, SCD1, and DGAT2 in firmicutes-treated mice.
136	4-week-old male C57BL/6 J mice		40% (wt) fructose diet for 7 weeks	Induction of fatty livers. Increased proteobacteria and colonic actinobacteria. Increased blood propionate and butyrate.
137	5-week-old male 129S6 mice		40% kcal HFD for 15 weeks	NAFLD caused by conversion of choline into methylamines by the microbiota. Lower plasma phosphatidylcholine and higher urinary excretion of methylamines.
138	8-week-old male C57BL/6 J mice		Methionine choline-deficient diet for 4 weeks	Increased NASH with liver fibrosis. Decreased *Bifidobacterium* and increased *Bacteroides*. Increased cholic acid, cholesterol, arachidic acid, and stearic acid in feces during NASH development.

### The role of microbiota-derived short-chain fatty acids (SCFAs) in NAFLD

How do fructose-mediated microbial population shifts affect the onset of NAFLD? One clinical study found a link between higher SCFA concentrations and an increased Firmicutes:Bacteroidetes ratio in obese and overweight individuals^138^. Increases in cecal SCFA concentrations have been found in obese mice with a concurrent similar increase in the Firmicutes:Bacteroidetes ratio, supporting the clinical findings^139,140^. While studies often report inconsistencies in the Firmicutes to Bacteroidetes ratios in obese and NAFLD patients, increased SCFA levels are consistently reported^138,141^. Another potential factor is microbiome diversity. One comparative study of 154 monozygotic and dizygotic twins that were incongruous for obesity found that the twin with obesity exhibited higher Firmicutes levels and an overall decrease in gut flora diversity^142^. It is thus possible that this diminished microbiome diversity, rather than specific microbial populations, contributes to NAFLD pathogenesis.

One interesting development in the field is the idea that excessive microbiome-derived SCFAs feed hepatic DNL, thereby triggering NAFLD. However, different SCFAs likely have different metabolic fates, with primarily acetate feeding DNL and propionate and butyrate feeding gluconeogenesis. In one clinical study, healthy fasted individuals who received acetate and propionate did not show increases in serum free fatty acids, presumably due to hepatic glucose production rather than DNL to compensate for fasting-induced hypoglycemia^143^. Another study found that infusion with SCFAs lowered free fatty acids while increasing serum TG and cholesterol levels^144^. Isotopic tracing of cecum-infused SCFAs confirmed a significant increase in the incorporation of SCFAs into glucose, cholesterol, and lipids^145^. Acetate, the most abundant SCFA, is preferentially utilized by hepatocytes via ACSS2 to fuel DNL. Zhao et al. found a twofold increase in acetate after acute fructose feeding in portal blood but not systemic blood, indicating that acetate is efficiently cleared by the liver^37^. The authors further found that depleting the microbiome using antibiotics or silencing hepatic ACSS2 markedly blocked fructose conversion into hepatic acetyl-CoA and fatty acids. Intriguingly, fructose-induced lipogenic gene expression was intact, consistent with the notion that proximal steps of fructolysis provide a signal to transcriptionally activate the DNL program. While involved in a range of various metabolic pathways, SCFAs also have an established role as signaling molecules via G-protein coupled receptors (GPCRs)^146,147^. In the liver, RGS5, a protein that suppresses these GPCRs, suppresses NAFLD/NASH pathology via downregulation of lipogenic genes and inflammatory cytokines in vivo^148^. Thus, targeted GPCR therapeutics may represent an alternative to chronic antibiotic usage in the treatment of NAFLD/NASH.

In contrast, some studies proposed the beneficial effects of SCFAs in reducing adiposity and restoring insulin sensitivity in obese mice^149,150^. Additionally, in contrast to what it does in the liver, acetate in the gut-brain axis has been shown to suppress appetite by upregulating γ-aminobutyric acid (GABA) activity in the hypothalamus and was thus flagged as a possible therapeutic for obesity^151^. The exact roles of propionate and butyrate in the liver are also under contention. Butyrate has been shown to robustly promote the mevalonate and fatty acid synthesis pathways by yielding intermediates such as HMG-CoA^152,153^. Propionate, on the other hand, inhibits the incorporation of acetyl-CoA into fatty acids without reducing cytosolic citrate levels, suggesting that it may compete with acetate for CoA and thereby suppress DNL and drive odd-chain fatty acid synthesis^154,155^. To make matters more complex, Weitkunat et al. found that the addition of both propionate and high amounts acetate in a long-term high-fat diet decreased the liver TG content and DNL gene expression, suggesting that SCFAs may play a protective role against steatosis^156^. Therefore, the role of SCFAs in NAFLD is likely context-dependent, and more systematic investigation is required.

### Effect of fructose on colon integrity

In addition to supplying lipogenic substrates via the microbiome, fructose itself causes intestinal damage to induce NAFLD/NASH. Fructose feeding has been shown to elevate plasma endotoxin levels, presumably due to increased oxidative stress and decreased intestinal junction integrity^157^. Bacterial endotoxins induce adipocyte lipolysis via Toll-like receptor 4 (TLR4) signaling and increase serum-free fatty acids^158^. Elevated free fatty acid levels have been linked to impaired insulin signaling^159^, and serum fatty acids that enter the liver can be incorporated into TGs, contributing to steatosis and insulin resistance^69,160,161^. A recent study further supported the notion that fructose-fed mice developed NAFLD/NASH phenotypes as a result of intestinal barrier deterioration and TLR signaling^162^. Antibiotic treatment blocked intestinal tight junction degeneration and lowered inflammatory hepatic cytokines and chemokines. Interestingly, antibiotics also suppressed DNL induction. Gut-mediated inflammation and endotoxemia may also explain NASH development. The progression of NAFLD into NASH has been described in the previous sections by the ‘multiple hit’ hypothesis, in which the first hits of lipid accumulation and insulin resistance are exacerbated by inflammation, leading to further hepatic injury. It is thus possible that fructose-induced gut permeability increases the liver’s susceptibility to these ‘second hits’ of systemic inflammation from serum endotoxins.

However, the human relevance of fructose-induced gut leakiness and microbial dysbiosis is still controversial. A recent clinical study showed that obese participants given a daily dose of 75 g of fructose over a course of a few weeks displayed no gut permeability or endotoxemia^126^. It is possible that the concentration of fructose administered in animal studies is beyond the concentrations received by typical human consumption, causing chronic and systemic changes. Conversely, these phenotypes may also be seen in humans if high fructose consumption proceeds long enough. Therefore, while understanding of the relationship between the microbiome, fructose, and hepatotoxicity in animal models has increased, more human data are necessary to draw the same conclusions regarding human NAFLD pathologies.

## Is dietary fiber, the major source of acetate produced by the microbiota, a friend or foe?

As described above, acetate is the preferential substrate of hepatic DNL. Even in the absence of fructose, copious acetate is produced by the microbial fermentation of dietary fibers. Dietary fibers, carbohydrate polymers with three or more carbohydrate units (primarily glucose or fructose), are naturally found in fruits, vegetables, and grains^163,164^. Some dietary fibers are resistant to digestive enzymes and therefore neither hydrolyzed nor absorbed until they reach the colon. Depending on their physicochemical characteristics, dietary fibers are subdivided into insoluble and soluble dietary fibers^165^. Insoluble fibers, such as cellulose and lignin, are barely fermented by the gut microbiota and thus have a fecal bulking effect^166^. In contrast, soluble fibers, such as inulin, β-glucan, pectins, and maltodextrins, are readily fermented to produce SCFAs^167^.

The concentration of SCFAs produced by the gut microbiota is largely influenced by the composition and type of dietary fibers in food^168^. In general, a diet containing more soluble fiber induces a higher concentration of acetate in fecal samples^169^, and this acetate readily feeds hepatic lipogenesis^170,171^. The roles of fiber-derived propionate and butyrate are less understood, but their presence in excessive amounts can induce insulin resistance^172^ or global gene alterations in colonocytes or hepatocytes, as butyrate can inhibit histone deacetylases (HDACs)^173–177^. Butyrate may thus provide a mode of epigenetic control. In colonocytes, the accumulation of butyrate has been shown to have a therapeutic effect as an HDAC inhibitor and suppresses colon tumorigenesis^178,179^. However, whether fiber-derived butyrate can reach millimolar concentrations to have any effects in hepatocytes is unclear.

Despite the potentially detrimental effect of dietary fibers through increased hepatic lipogenesis and perturbation of signaling pathways, certain types of dietary fibers reduce the risk of metabolic diseases^180,181^. In animal studies with a fructose-rich diet, supplementation with inulin significantly reduced blood TG and cholesterol levels^182,183^. Similar effects were also observed in clinical studies. In a dietary intervention study, increasing inulin intake reduced blood TG levels and fat mass in obese children^184^. Twelve healthy participants who consumed cereal containing high amounts of inulin for four weeks showed significantly lower plasma TG and cholesterol levels than the control group^185^.

To determine the causal relationship between fiber fermentation and improved metabolism, several studies have focused on the impact of an SCFA-enriched diet. Supplementation with propionate and butyrate improved glucose homeostasis in both clinical and animal studies^149,186^. On the other hand, in type 2 diabetic rats, acetate supplementation reduced hepatic TG accumulation and improved glucose tolerance by decreasing SREBP-1^187^. SCFA supplementation has also been implicated in the prevention of CVD. Acetate significantly reduced blood pressures, cardiac fibrosis, and left ventricular hypertrophy in a mouse model of CVD induced with deoxycorticosterone, and a high-fiber diet generated consistent improvement^188^. This protective action of dietary fibers and acetate likely involves downregulation of CVD-associated genes, such as early growth response protein 1 (Egr1).

However, other recent studies have indicated that dietary fibers can be detrimental. In a randomized clinical study, Chambers et al. compared the effect of inulin or inulin-propionate ester consumption in NAFLD patients. In contrast to the authors’ prediction, after 42 days of 20 g/day inulin supplementation, the subjects showed significantly increased intrahepatocellular lipid accumulation. The provision of inulin-propionate ester did not have this effect. Given that NAFLD patients exhibit high levels of hepatic DNL, the authors speculated that acetate derived from the high-inulin diet provided excessive carbon for increased DNL, while propionate supplementation suppressed it by competing with acetate for CoA^189^. A high-inulin diet was also shown to worsen liver cancer. Feeding TLR5-deficient mice a 7.5% inulin diet caused cholestasis, hepatic inflammation, and hepatocellular carcinoma^190^. Germ-free or antibiotic-treated mice did not show these phenotypes, indicating that microbiota dysbiosis was involved. This intriguing idea of context-dependent fiber effects on lipogenesis and liver pathology merits further investigation.

To conclude, despite the widespread belief in the beneficial effects of dietary fibers, the composition of dietary fibers and health status of the individual (e.g., insulin resistance, NAFLD, microbiota dysbiosis) are likely critical factors that determine the impact of dietary fibers on metabolic health. This notion supports the increasingly popular notion of personalized medicine and diet. Future studies using isotope tracing and metabolomics to define the metabolic fates of various dietary fibers and their byproducts in diverse pathophysiological contexts will expand our understanding of the link between fibers, their fermentation products, and NAFLD onset.

## Concluding remarks

A recent alarming increase in NAFLD patients and patients with associated detrimental metabolic diseases has sparked extensive research in the field. Numerous dietary and genetic animal models have been prepared to recapitulate the human pathology, and the use of these models has identified several new metabolic pathways involved in the disease process. Among them, increased hepatic lipogenesis driven by a high-carbohydrate diet and microbial metabolism/dysbiosis have been repeatedly reported in NAFLD. Accordingly, targeting hepatic lipogenesis via inhibition of a specific lipogenic enzyme or limiting the supply of lipogenic carbon substrates has been performed in clinical trials and achieved some promising results. In addition to the further development of therapeutic strategies, a more detailed understanding of the complex mechanisms of NAFLD pathogenesis in various genetic and dietary backgrounds (e.g., >100 outbred mouse strains or large-scale human dietary intervention studies) will explain why some people are more susceptible to NAFLD than others. This will also expedite the development of personalized dietary guidelines and pharmaceutical interventions. Finally, identifying accurate and early diagnostic markers of NAFLD in the blood and/or fecal samples is another crucial future research avenue.
